# Behavioral differentiation during the AIGC image generation waiting period: effects on creativity in conceptual design

**DOI:** 10.3389/fpsyg.2026.1924155

**Published:** 2026-10-12

**Authors:** Ziliang Zhu, Jiang Xu

**Affiliations:** Pan Tianshou College of Architecture, Art and Design, Ningbo University, Ningbo, China

**Keywords:** AIGC generation waiting period, cognitive load, conceptual design, creativity, task relevance

## Abstract

**Introduction:**

Artificial intelligence-generated content (AIGC) offers new ways to generate inspiration for conceptual design, yet limited attention has been paid to the waiting period between prompt submission and AI image output. This study examined behavioral differentiation during this interval and investigated how the task relevance and cognitive load of waiting-period behaviors affect creativity in conceptual design.

**Methods:**

Three experiments were conducted with design students. Experiment 1 identified and classified core waiting-period behaviors (*N* = 30); Experiment 2 measured the cognitive load of representative behaviors using the NASA-TLX (*N* = 24); and Experiment 3 employed a behavior-based 2 × 2 conceptual framework (*N* = 60), in which four representative waiting-period behaviors were selected to represent different combinations of task relevance (high/low) and relative workload (higher/lower), and their creative performance was compared.

**Results:**

The results showed that the AIGC generation waiting period was not a passive interval but involved multiple identifiable behavioral patterns. Creative performance differed across the four representative behavioral conditions. Specifically, the overall creativity score in the GP condition was significantly lower than the scores in the RS, DB, and RB conditions, whereas no significant differences were observed among the latter three conditions. These findings further suggest that the subsequent influence of waiting-period behaviors on creativity may depend not only on the level of cognitive demand but also on whether cognitive resources remain directed toward the ongoing creative task.

**Discussion:**

This study further interprets AIGC generation waiting-period behaviors from three interrelated functional perspectives: task continuity, cognitive expenditure, and temporary disengagement/incubation. This functional account provides an empirical basis for understanding the cognitive role of the AIGC generation waiting period and for optimizing AI-assisted conceptual design workflows.

## 1 Introduction

Conceptual design is the core activity of design, serving as a crucial bridge connecting human needs, creative ideas, and product realization. It occurs in the early stage of product development but determines 70% to 85% of the product's life cycle cost, serving as the foundation for subsequent design, production, operation and maintenance stages (French et al., 1985; Wang et al., 2023). The conceptual design phase requires designers to translate open-ended requirements into innovative solutions; it is a critical stage where design creativity begins to emerge, placing sustained and substantial cognitive demands on designers' existing knowledge structures and working memory (Myrup Andreasen et al., 2015). Traditional methods such as brainstorming, sketching, and collaborative discussion have long served as fundamental approaches to idea generation and problem solving in this phase. However, designers are often limited by personal experience, habitual thinking patterns, and the subjectivity of ideation (Fu et al., 2026). These constraints may adversely affect design efficiency and creative output.

Advances in generative language models have brought artificial intelligence-generated content (AIGC) to the forefront of research and industrial attention. It typically refers to a subset of artificial intelligence technology that uses machine learning models (such as neural networks) to identify patterns in existing training data, thereby generating new content—including text, images, and models (Fui-Hoon Nah et al., 2023). With its powerful capabilities in creative integration and content generation, AIGC has demonstrated tremendous potential for enhancing creative outcomes during the conceptual design phase and stimulating design creativity (Fang et al., 2025; Rezwana and Maher, 2023), and is emerging as a new tool for boosting design productivity. By drawing on advanced algorithms and machine learning techniques, AIGC can process and integrate large-scale data within a short period of time (Foo et al., 2025), extract user needs and design-related semantics from such data (Chen et al., 2024), and rapidly produce textual or visual materials that provide inspiration for design ideation (Wan and Lu, 2023; Yun et al., 2022). In the early stages of design, AIGC can function as an auxiliary tool for sketching and ideation (Arias-Rosales, 2022). It may also act as a creative partner for designers by providing multiple outputs that can be compared in parallel to support concept visualization (Oh et al., 2019; Ye et al., 2024), and can further be used for the analysis and evaluation of design proposals (Camburn et al., 2020). Overall, AIGC supports rapid iteration by accelerating the cycle of generation, comparison, and screening (Wang et al., 2024), thereby improving design efficiency and, in many contexts, extending the creative capacity of designers (Bouschery et al., 2023; Zeng et al., 2019).

Although AIGC can substantially expand creative potential, it also introduces non-negligible processing latency (Ding et al., 2022). In image-generation tasks, the interval between prompt submission and the return of the final image typically ranges from several seconds to several minutes, depending on factors such as sampling steps, operating mode, concurrent request volume, and deployment architecture (Rombach et al., 2022). Studies on users' perceptions of generation waiting time further suggest that users do not necessarily resist waiting during the generation process. Within a moderate range, they may instead regard such waiting as an inherent part of the creative process (Lee et al., 2025; Tan et al., 2026). Generation waiting should therefore not be understood merely as a negative cost. It can be actively leveraged, or even deliberately introduced, as a creative buffer that allows users to pause, anticipate, and prepare for subsequent ideation (Liu et al., 2024). Accordingly, the generation waiting period is not only a temporal interval created by technical latency. It may also constitute a process window that influences users' thinking, behavioral choices, and subsequent creative states. However, existing research has mainly discussed generation latency in terms of waiting duration, generation cues, or user perception, leaving the specific behavioral differentiation within the waiting period and its consequences for design cognition insufficiently examined.

In AIGC-mediated conceptual design, interaction is often organized as a recurrent cycle of prompting, generation, output, and evaluation (Wu et al., 2025). The brief interval between the prompt and the AI's output (hereinafter referred to as the “generation waiting period”) is not a period of inactivity; during this wait, designers engage in a variety of activities, such as sketching, searching for references, refining prompts, reflecting on the task, or taking a break to relax and entertain themselves (Liu et al., 2024). These behaviors vary in their degree of task relevance and cognitive load. Some directly maintain the continuity of current design cues, whereas others temporarily move designers away from the task into a less task-relevant or more relaxed state. Bodily behavior can also be understood as a non-verbal indication and expression of implicit interaction (Zhao et al., 2013). By shaping attention allocation, information integration, and incubation processes (Ritter and Dijksterhuis, 2014), such behaviors may further influence how designers interpret AI-generated outputs and how they proceed with subsequent re-creation. Existing research has largely focused on prompting strategies or the analysis of generated outcomes (Oppenlaender et al., 2025; Oppenlaender, 2024), leaving designers' behaviors during the generation waiting period and their potential cognitive consequences insufficiently examined. This study therefore focuses on the AIGC generation waiting period as a critical moment in human–AI collaboration. It examines behavioral variation and cognitive effects within this period, with particular attention to how the task relevance and cognitive load of waiting-period behaviors influence designers' creativity.

Cognitive load refers to the burden placed on the cognitive system by a specific task (Paas and Van Merriënboer, 1994), that is, the total mental effort expended in working memory; it significantly influences creative output in design tasks (Chandrasekera et al., 2024). Cognitive Load Theory (CLT) categorizes cognitive load into intrinsic load, extraneous load, and germane load. It emphasizes that task performance can be improved when extraneous load is reduced while cognitive resources relevant to the task are preserved (Sweller et al., 1998; van Merriënboer and Sweller, 2005). In the context of the generation waiting period, different behaviors may vary not only in their relevance to the ongoing design task but also in the extent to which they require or consume cognitive resources. Cognitive load therefore provides an important theoretical perspective for explaining how waiting-period behaviors may influence subsequent design cognition and creative performance.

Creativity is commonly understood as the capacity of individuals or groups to solve problems and generate novel yet appropriate concepts, products, or services (Amabile, 1983; Sternberg and Lubart, 1996). It is also sensitive to external conditions (Amabile et al., 1996), with cognitive load being one of the important factors that shape creative performance (Hernandez Sibo et al., 2024). High cognitive load is generally considered detrimental to creativity because it consumes cognitive resources that are needed for divergent thinking, a core component of creative ideation (Chandrasekera et al., 2024). However, an insufficient level of cognitive load may also fail to stimulate creative thinking effectively (Baer and Oldham, 2006). Previous studies have shown that cognitive underload in learning or task performance contexts can lead to reduced performance (Bannert, 2002; Svensson et al., 1997). Cognitive demand may therefore influence creative processing, but differences in load level alone are insufficient to explain the effects of different waiting-period behaviors, it is also necessary to consider whether the cognitive resources consumed remain directed toward the ongoing creative task.

In addition, creativity in the design process does not rely solely on continuous conscious thought (Sio and Ormerod, 2009). Research on the incubation effect suggests that, after an initial period of problem processing, a moderate temporal interval may help individuals temporarily move beyond the constraints of existing ideas. This interval may support creative problem solving through mechanisms such as unconscious processing, problem re-representation, and the restoration of attentional resources (Ritter and Dijksterhuis, 2014; Ghiselin, 1952; Wallas, 1926). In temporal terms, the “generation waiting period” in AIGC-assisted design shares certain features with the incubation period. Both occur after the initial processing of a problem, and both provide designers with a brief window in which they are temporarily released from the immediate demand to produce an output. The difference is that the AIGC generation waiting period is not a pure task suspension (Karimi et al., 2020), as designers may still continue to engage in different task-related or task-unrelated activities. Therefore, its effects cannot be explained solely by whether waiting occurs, but also require consideration of the nature of the specific behaviors performed during the waiting period.

The incubation period likewise does not imply a complete cessation of cognitive activity. Previous research suggests that the cognitive demands of activities undertaken during an incubation interval can influence subsequent creative performance: cognitively demanding intervening tasks tend to attenuate the incubation effect, whereas low-demand activities may, in some contexts, be more conducive to subsequent creativity than complete rest (Sio and Ormerod, 2009; Baird et al., 2012). Incubation theory and Cognitive Load Theory therefore provide two complementary perspectives on the AIGC generation waiting period. The former highlights the potential for temporary disengagement from immediate output demands to facilitate fixation release and attentional restoration, whereas the latter emphasizes the consumption of limited cognitive resources by activities performed during the waiting period. From this functional perspective, the potential cognitive roles of AIGC generation waiting-period behaviors can be understood in terms of three interrelated dimensions: task continuity, referring to the maintenance of current design goals and relevant cues; cognitive expenditure, referring to the consumption of limited cognitive resources by waiting-period activities; and temporary disengagement/incubation, referring to a temporary reduction in direct task processing that may provide opportunities for attentional restoration or fixation release. On this basis, the present study further proposes that the cognitive consequences of the AIGC generation waiting period may depend not only on the magnitude of cognitive demand but also on whether the cognitive resources consumed remain aligned with the ongoing creative task.

Taken together, existing research indicates that creativity during the conceptual design phase is a multidimensional, interactive outcome that is shaped not only by an individual's knowledge structure and cognitive resources but also by the combined influence of task constraints, contextual factors, and collaborative interactions. Meanwhile, studies on AIGC-assisted design have primarily examined prompting strategies, generated outputs, and the effects of different human–AI collaboration modes on design workflows. Comparatively less attention has been paid to the temporal structure of the design process and to the cognitive dynamics that unfold within specific process intervals. Against this background, the present study investigates behavioral variation during the AIGC generation waiting period and examines its influence on creativity. Through three experiments, this study addresses the following research questions: First, what typical behavioral patterns can be identified during the waiting period of AIGC-based image generation? Second, do these waiting-period behaviors differ in terms of task relevance and cognitive load? Third, how do waiting-period behaviors characterized by different levels of task relevance and cognitive load influence subsequent creativity in conceptual design? Based on the systematic examination of the aforementioned issues, this study provides theoretical and empirical support for the interactive design and practical application of AIGC.

## 2 Experiment 1: Identification and classification of behaviors during the AIGC generation waiting period

### 2.1 Methods

#### 2.1.1 Participants

Thirty undergraduate and graduate students from design-related programs were recruited for this experiment (age: *M* = 23.57, *SD* = 0.97), with a gender ratio of approximately 1:1. Eligibility required participants to have used Midjourney at least once before the experiment, thereby excluding first-time users with no prior interaction experience and minimizing potential disruption associated with initial familiarization with the tool. The experiment was conducted at Ningbo University. All participants were required to sign informed consent forms before the commencement of the experiment and participated voluntarily, receiving a specified compensation upon the conclusion of the study. Experiment 1 was primarily exploratory, aiming to identify and classify naturally occurring waiting-period behaviors, accordingly, no sensitivity analysis was conducted for a prespecified effect. According to APA ethical guidelines, the ethics committee of the institution where the first author is affiliated has approved this research.

#### 2.1.2 Task relevance assessment

To ensure fairness and accuracy in the scoring process, the evaluation of task relevance was conducted by a panel of two design experts. Each expert had over five years of experience in design practice or design research. The experts used a seven-point Likert scale (with one indicating low task relevance and seven indicating high task relevance) for scoring. The theoretical midpoint for a single dimension is four points, and the combined theoretical midpoint for all four dimensions is 16 points. Therefore, a total score of 16 was used as the threshold for classifying task relevance. For each category of waiting-period behavior, the final score was calculated as the mean of the scores assigned by the two experts.

Before the expert ratings, the assessors were provided with unified behavioral definitions, descriptions of the scoring dimensions, and scoring criteria. The task relevance of participants' waiting-period behaviors was assessed across four dimensions: semantic relevance, goal orientation, tool reliance, and attentional continuity. Semantic relevance refers to the extent to which the visual or textual information involved in a behavior overlaps in meaning with the current design theme (Mayer and Moreno, 2003). A higher level of semantic relevance indicates that the visual or textual information encountered during the behavior is more closely aligned with the design task. Goal orientation measures whether the behavior reflects a clear motivation to advance the design task or support decisions related to design output (Locke and Latham, 2002). This dimension serves as a key indicator for distinguishing core cognitive operations from peripheral disengagement behaviors. A higher score indicates that the behavior more clearly reflects an intention to move the task forward. Tool reliance mainly examines whether the software or hardware medium with which the participant interacts belongs to the core design workflow. Since cognitive processes are widely distributed across the external physical and digital tools used by individuals (Hollan et al., 2000), sustained reliance on core tools is an important indicator of maintaining a task-relevant cognitive context. A higher score indicates that the participant's cognitive processing is more deeply embedded in the design workflow. Attentional continuity refers to whether participants consistently maintain focus on the current task and keep relevant information in a processable state (Engle, 2002).

#### 2.1.3 Experimental design

Experiment 1 simulated an AI-assisted conceptual design context to observe and document participants' behaviors during the generation waiting period. The task relevance of different categories of waiting-period behaviors was further assessed across four dimensions: semantic relevance, goal orientation, tool reliance, and attentional continuity.

#### 2.1.4 Materials

The experiment was conducted in a quiet, separate observation room (see Figure 1). The experimental materials and equipment included a laptop with a stable internet connection, standard A4 paper, and sketching pens. Midjourney was used as the AIGC tool for generating inspirational images for design. To document participants' behaviors during the generation waiting period, screen recording was used together with video recording from two camera angles: Camera A was used to capture participants' facial expressions and body posture, while Camera B was used to record their sketching behavior in the desktop area. After completing the task, participants submitted their final conceptual design proposals.

**Figure 1:**
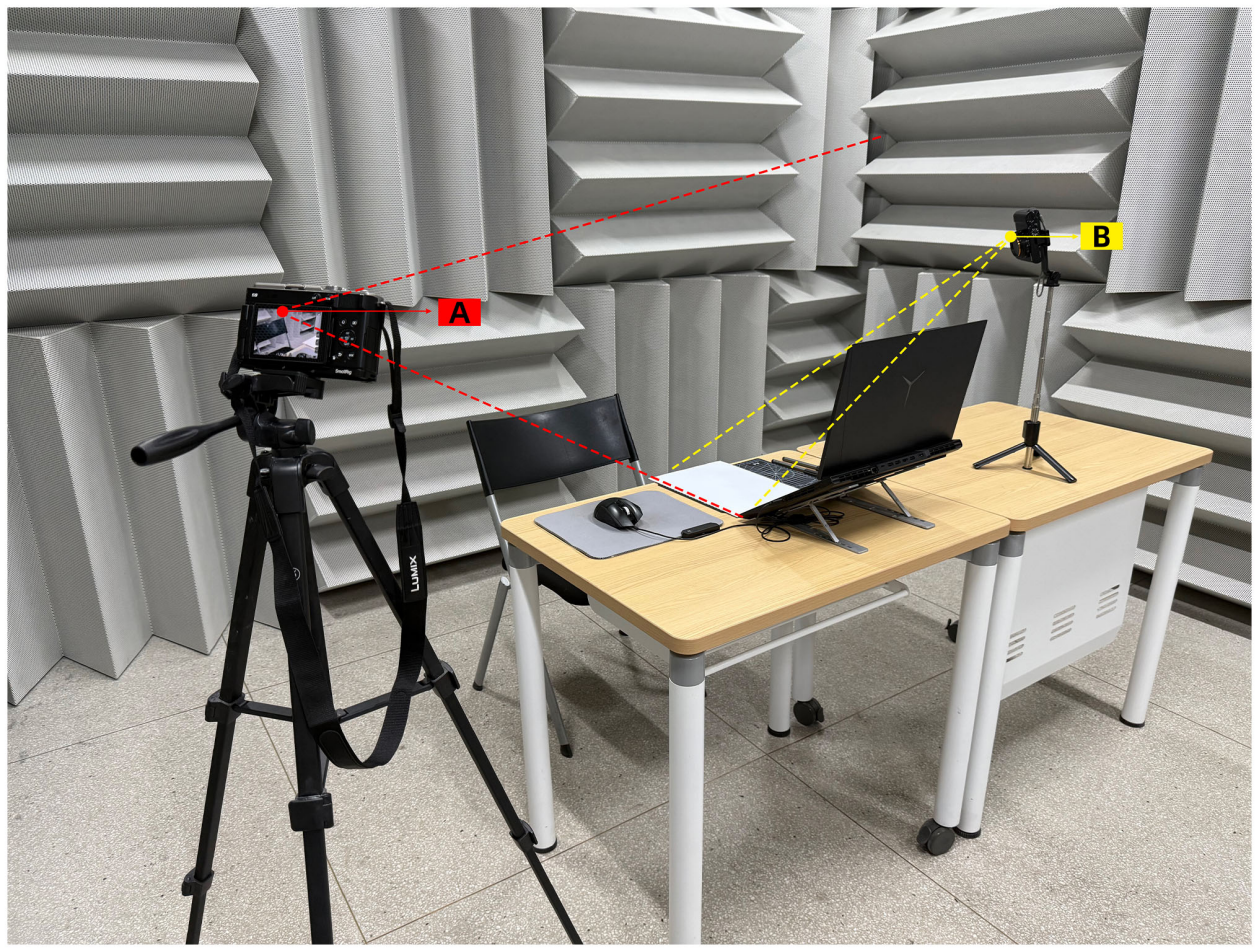
Experimental setup. **(A)** Body and facial tracking camera; **(B)** Desktop operation tracking camera.

#### 2.1.5 Procedure

The procedure for Experiment 1 was as follows (see Figure 2). Participants completed the experimental task in a simulated conceptual design context. They first read the experimental instructions and completed a pre-task questionnaire. Before the formal task, participants received a 10-min training session covering the basic conceptual design process and the use of Midjourney to generate conceptual design proposals. They then received a conceptual design brief—to develop a portable cup tailored for urban commuting. This topic was closely related to everyday life and did not require advanced engineering knowledge, which helped lower the threshold for task comprehension and ensured the feasibility of the design activity.

**Figure 2:**
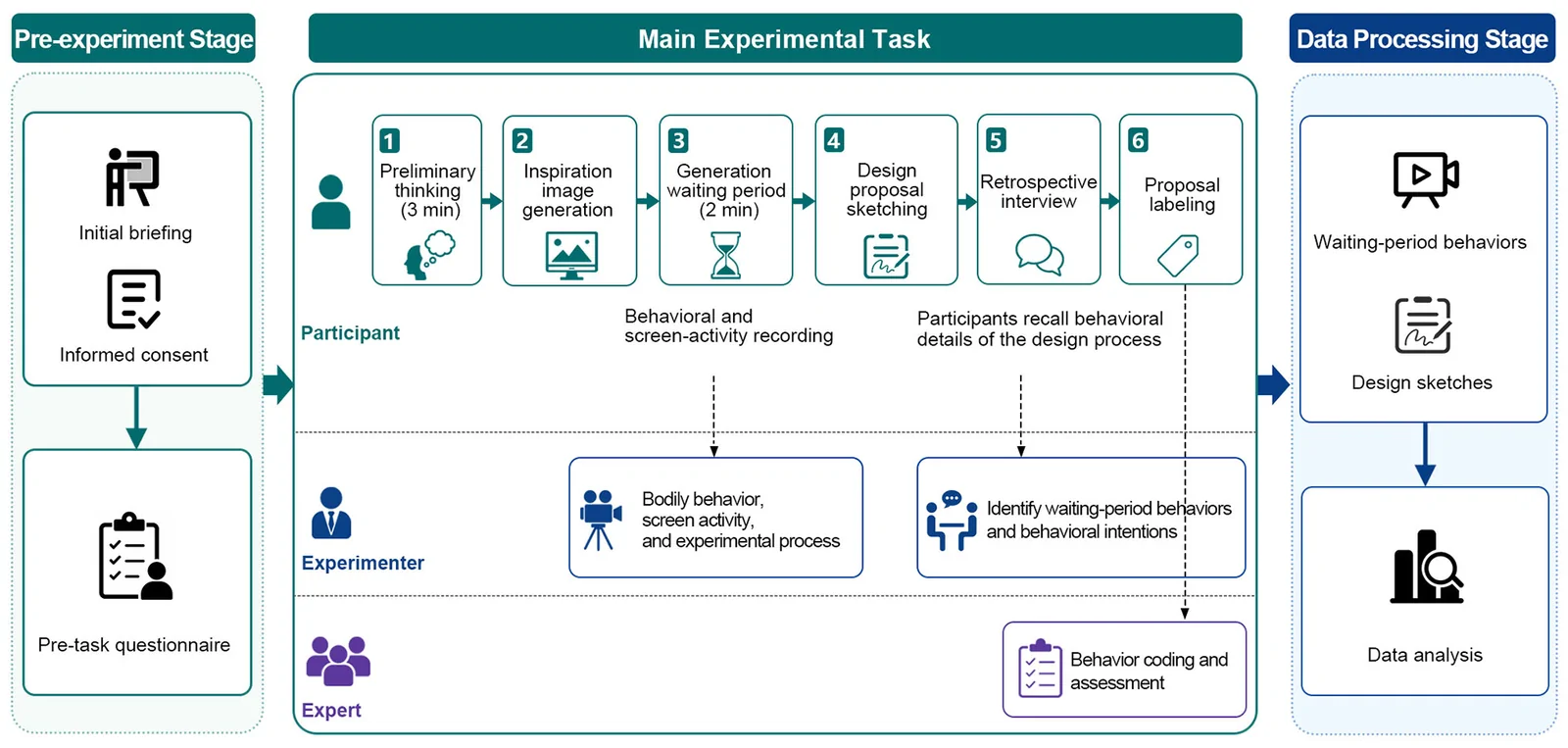
Procedure overview of Experiment 1.

Before engaging with the main design task, participants were allocated a 3-min reflection phase to internalize the design brief and establish an initial conceptual direction. Following this, they used Midjourney to generate inspirational imagery. For each iteration, the interval between prompt submission and image output was defined as the generation waiting period. To capture waiting-period behaviors as they naturally occurred, participants were not given additional instructions regarding what they should do during this interval. Each participant could complete up to six full image-generation iterations. When participants considered the generated images sufficient to support subsequent ideation, they used paper and pen to represent their final design proposal. After the task was completed, the experimenter conducted a brief retrospective interview based on the experimental records and design sketches to help confirm the main behaviors performed during the generation waiting period and the intentions underlying those behaviors.

#### 2.1.6 Data analysis

Based on screen recordings and dual-camera behavioral video recordings, the principal investigator employed a method combining data-driven behavioral category development with dual-indicator screening to identify candidate behavioral categories from the raw recordings and pinpoint core behaviors during the waiting period (Jia et al., 2024) (see Figure 3). Following the principle used in design-process research to segment continuous activity at meaningful behavioral changes (Suwa et al., 1998), a new behavioral segment was marked whenever a substantive change occurred in the participant's primary action, object of interaction, or observable activity content. A minimum duration threshold of 5 s was applied in conjunction with the identification of brief and transitional actions during preliminary review of the video recordings (Kirby et al., 2017). Only segments lasting at least 5 s and exhibiting a clearly identifiable activity purpose were retained as valid units of analysis. Without imposing a predefined coding scheme, the lead researcher developed 46 initial behavior labels based on the primary action, object of interaction, and activity content directly observable within each segment. When the purpose of an activity could not be determined from the dual-camera behavioral videos alone, the segment was further cross-checked against the screen recording and the retrospective interview conducted after task completion; segments that remained unclassifiable after this cross-check were labeled “uncodable” and excluded. Through three rounds of iterative comparison and consolidation based on similarities in these characteristics, the 46 initial labels were grouped into 17 candidate behavior categories. Their operational definitions, coding boundaries, and final core-behavior designations are provided in Supplementary Table S1. The lead researcher then developed a coding manual specifying the name, code, operational definition, observable coding criteria, inclusion and exclusion criteria, and boundary rules for each category.

**Figure 3:**
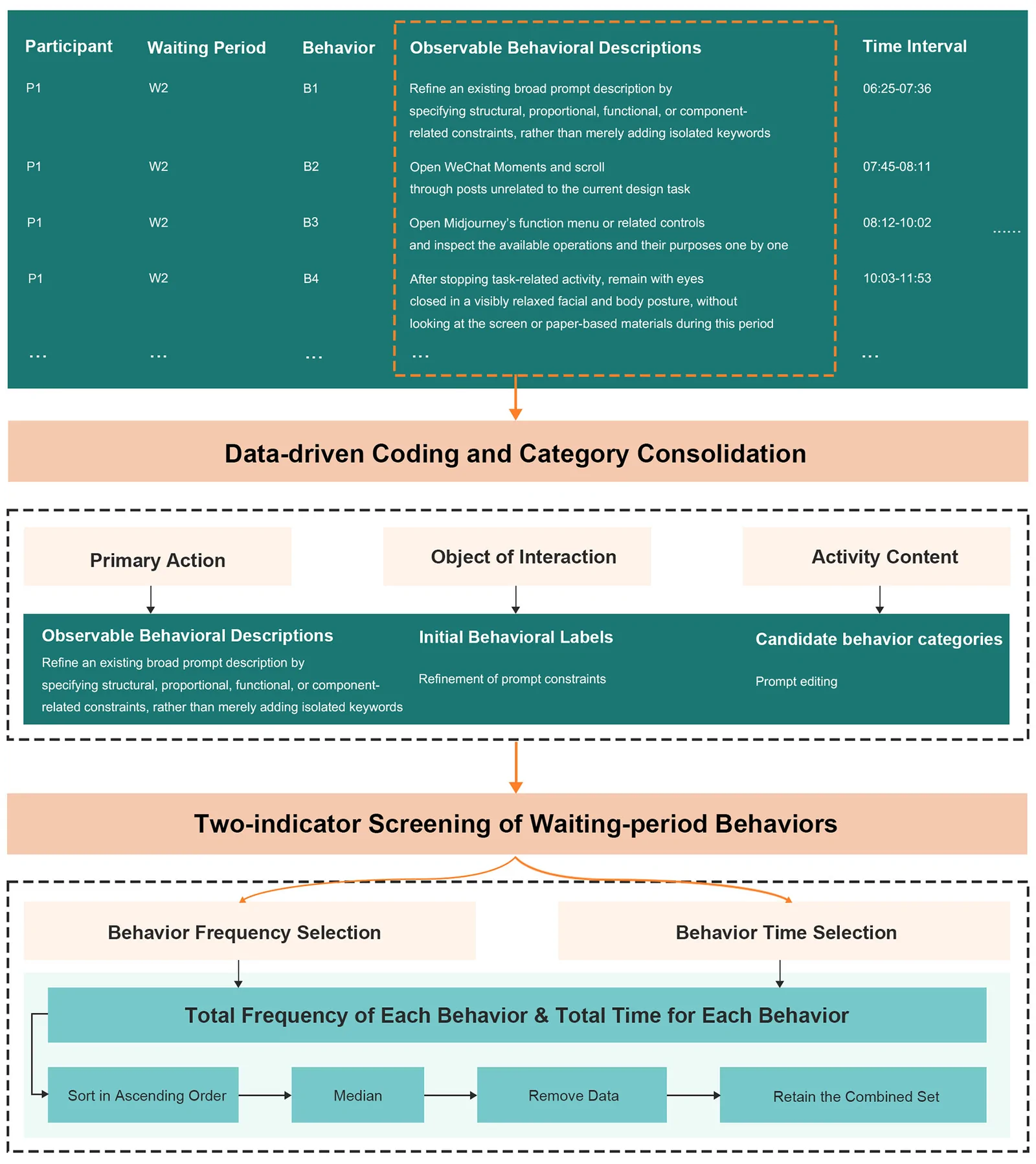
Two-indicator behavioral screening method.

After the coding manual was finalized, two coders with experience in design research completed two structured training sessions totaling approximately 4 h, followed by trial coding and rule calibration using three video recordings with a combined duration of approximately 60 min. During the formal coding phase, the primary coder coded all video clips, while the second coder independently recoded a representative subsample of approximately 20 percent without knowledge of the primary coder's results. Cohen's κ = 0.82, indicating a high level of inter-coder agreement. The researchers discussed and cross-checked the coded content where discrepancies existed between the two, and after reconciling the differences, they used the consolidated data for subsequent analysis. The total occurrence frequency and cumulative duration of each of the 17 candidate behavior categories were then calculated, after which the dual-indicator behavioral screening method was applied. Categories falling below the respective median values for both occurrence frequency and cumulative duration were excluded as low-frequency, short-duration behaviors, whereas all remaining categories were retained in the core behavior pool.

### 2.2 Results

After data cleaning, 90 valid video recordings were retained, with a combined duration of approximately 30 h. Across all recordings, 223 behavioral segments lasting at least 5 s were identified. The behavioral purpose of three segments remained unclear after cross-checking the screen recordings, dual-camera behavioral recordings, and retrospective interview records; these segments were therefore labeled “uncodable” and excluded, leaving 220 valid behavioral segments for subsequent analysis. Across the 17 candidate behavior categories, the median total occurrence frequency was 10, and the median cumulative duration was 416 s. Application of the dual-indicator behavioral screening method ultimately retained 11 core behavior categories, whose names and codes are presented in Table 1.

**Table 1:** Behavior codes.

Number	Name	Code
1	Reference information retrieval	IR
2	Prompt editing	PE
3	Generation progress monitoring	GP
4	Social messaging interaction	SM
5	Design brief review	DB
6	Rest break	RB
7	Rapid sketching	RS
8	Idle waiting	IW
9	Unstructured web switching	UW
10	Entertainment content browsing	EB
11	AI prompt retrieval	AP

Two design experts rated the task relevance of the 11 core behaviors. The ratings classified 5 of the 11 waiting-period behaviors as highly task-relevant and the remaining 6 as low in task relevance. The detailed classification results are presented in Figure 4. To examine the sensitivity of the classification results to the theoretical threshold, the total score classification threshold was adjusted to a range of 14–18 points, and the data were reclassified. The results showed that all behavioral classifications remained unchanged at a threshold of 15–18 points. When the threshold was lowered to 14 points, only GP (*M* = 14.50) exhibited a change in classification. Therefore, the overall classifications are relatively stable near the theoretical midpoint, but GP is relatively sensitive to lower thresholds. Internal consistency across the four rating dimensions was further examined (see Table 2), and inter-rater agreement between the two experts was assessed (see Table 3). Cronbach's α was 0.960, indicating that the four rating dimensions demonstrated a high level of internal consistency. The high α value also suggests a strong commonality among the four dimensions; therefore, this study treats them as theoretically distinguishable yet interrelated aspects of evaluation under the overarching construct of “task relevance,” rather than as independent constructs. The intraclass correlation coefficient analysis showed that the single-measure ICC(A,1) was 0.939 and the average-measure ICC(A,K) was 0.969, suggesting a high level of agreement between the two experts.

**Figure 4:**
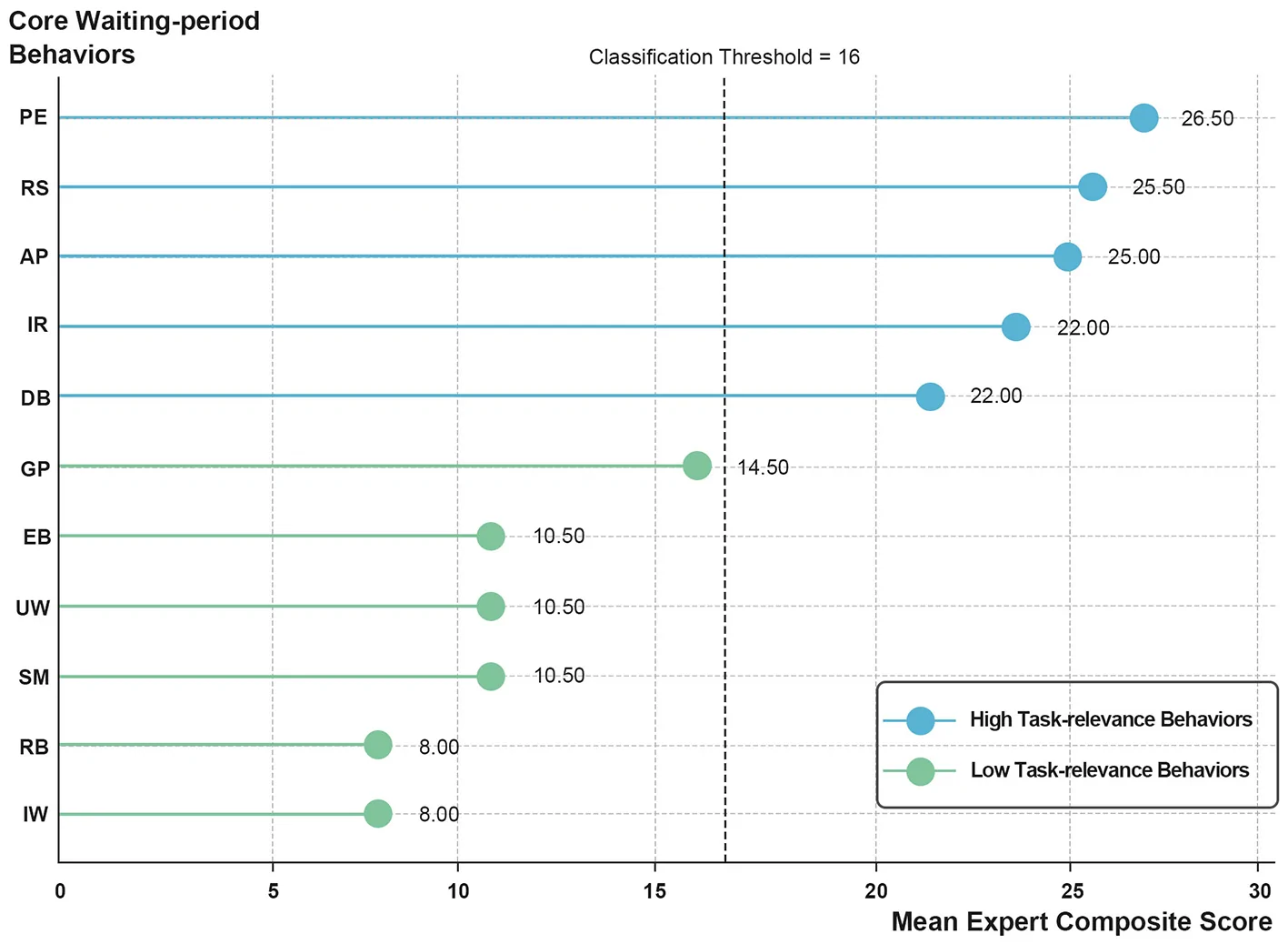
Expert scoring of core behavioral task relevance.

**Table 2:** Internal consistency of the task-relevance rating dimensions.

Cronbach's α	Number of rating dimensions
0.960	4

**Table 3:** Inter-rater agreement of expert ratings.

ICC type	Model	Agreement type	ICC	95% CI
Single-measure ICC, ICC(A,1)	Two-way mixed-effects model	Absolute agreement	0.939	0.789–0.983
Average-measure ICC, ICC(A,k)			0.969	0.882–0.992

### 2.3 Interim discussion

The findings of Experiment 1 indicate that the waiting period during AIGC image generation is not merely a passive phase but involves a variety of identifiable and classifiable behavioral patterns. After filtering by frequency and duration, this study identified 11 core waiting-period behaviors, establishing a preliminary classification system for waiting-period behaviors in the current AIGC-assisted conceptual design context, which provides a foundation for selecting representative behaviors and constructing conditions in subsequent experiments. Further expert evaluations revealed clear differences in task relevance among the various waiting-period behaviors, which were categorized into two groups: high task relevance and low task relevance. This indicates that waiting-period behaviors are not homogeneous activities but rather types of cognitive behaviors characterized by varying degrees of task engagement.

## 3 Experiment 2: subjective workload measurement of representative behaviors during the AIGC generation waiting period

Building on the findings from Experiment 1, Experiment 2 selected representative waiting-period behaviors to further measure the differences in subjective workload they induce during the waiting period of AIGC image generation.

### 3.1 Methods

#### 3.1.1 Participants

An additional 24 graduate students from design-related programs at Ningbo University, China, were recruited for Experiment 2 (age: *M* = 23.75, *SD* = 1.62). The gender ratio was nearly balanced at a ratio of approximately 1:1. Eligibility required participants to have basic sketching ability, defined as the ability to communicate design ideas clearly through sketches. This ability was confirmed either through a review of participants' previous design sketches or through confirmation by a faculty member in design. The experiment was conducted at Ningbo University. All participants were required to sign informed consent forms before the commencement of the experiment and participated voluntarily, receiving a specified compensation upon the conclusion of the study. As no a priori power analysis was conducted, a sensitivity analysis based on the achieved sample size indicated that, with α = 0.05 and statistical power of 0.80, the repeated-measures design (*N* = 24) had a minimum detectable effect size of Cohen's *f* = 0.278, indicating adequate sensitivity to moderate or larger effects. According to APA ethical guidelines, the ethics committee of the institution where the first author is affiliated has approved this research.

#### 3.1.2 Subjective workload assessment

This study used the Raw NASA Task Load Index (Raw TLX) to assess participants' subjective workload across different waiting-period behavior conditions. The Raw TLX is calculated without weighting the individual dimensions, with the arithmetic mean of the raw ratings across all six dimensions serving as the overall subjective workload score (Hart, 2006). All six dimensions were rated on a 0–100 scale. Higher scores on mental demand, physical demand, temporal demand, effort, and frustration indicated greater workload on the corresponding dimension, whereas the performance dimension was scored such that higher scores represented poorer self-rated performance (Hart and Staveland, 1988). Accordingly, higher scores across all six dimensions consistently represented greater subjective workload. For each behavior condition, the overall Raw TLX score was calculated as the arithmetic mean of the six dimension scores—mental demand, physical demand, temporal demand, performance, effort, and frustration—as follows:


$$\begin{array}{l} \text{RTLX }=\;\frac{\text{MD }+\;\text{PD }+\;\text{TD }+\;\text{P }+\;\text{EF }+\;\text{FR}}{6} \end{array}$$

#### 3.1.3 Experimental design

Experiment 2 employed a single-factor within-subjects design, with waiting-period behavior as the independent variable and the NASA-TLX subjective workload score as the dependent variable. Based on the 11 core behavior categories identified in Experiment 1, Experiment 2 selected behavioral conditions according to three rules: the selected set must collectively cover both high- and low-task-relevance behaviors; they must be capable of being performed independently, continuously, and unambiguously by participants under a standardized experimental procedure; their behavioral boundaries must be clearly defined, while additional variability caused by interaction partners, open-ended behavioral paths, or externally generated systems should be minimized. Based on these criteria, PE, RB, RS, IW, IR, EB, DB, and GP were included. SM relies on immediate responses from interaction partners; the browsing paths and content of UW are difficult to standardize and may overlap with IR or EB; and AP relies on dynamic outputs from an external AI system and partly overlaps with PE in prompt processing. Therefore, these three behaviors were not included in Experiment 2.

#### 3.1.4 Materials

To ensure consistency in behavioral induction, standardized physical and digital materials were configured for each waiting-period behavior in Experiment 2. For example, in the PE condition, participants added, removed, revised, or refined keywords based on a standardized initial prompt; in the GP condition, participants monitored a standardized generation state and progress display over a fixed waiting period through a custom-built AIGC simulation interface. The remaining behavior conditions used standardized task materials or behavioral instructions consistent with their corresponding operational definitions.

#### 3.1.5 Procedure

The procedure for Experiment 2 was as follows (see Figure 5). The experimenter guided participants into the “portable cup conceptual design” task scenario and provided training on how to complete the NASA-TLX scale. During the formal testing phase, each participant underwent eight simulated image-generation waiting periods, each lasting a fixed 120 s. During each waiting period, participants performed designated actions in the order predetermined by an 8 × 8 balanced Latin square. Immediately after each waiting period, participants were required to complete the NASA-TLX scale via a sliding scale on a tablet to record their immediate subjective workload levels.

**Figure 5:**
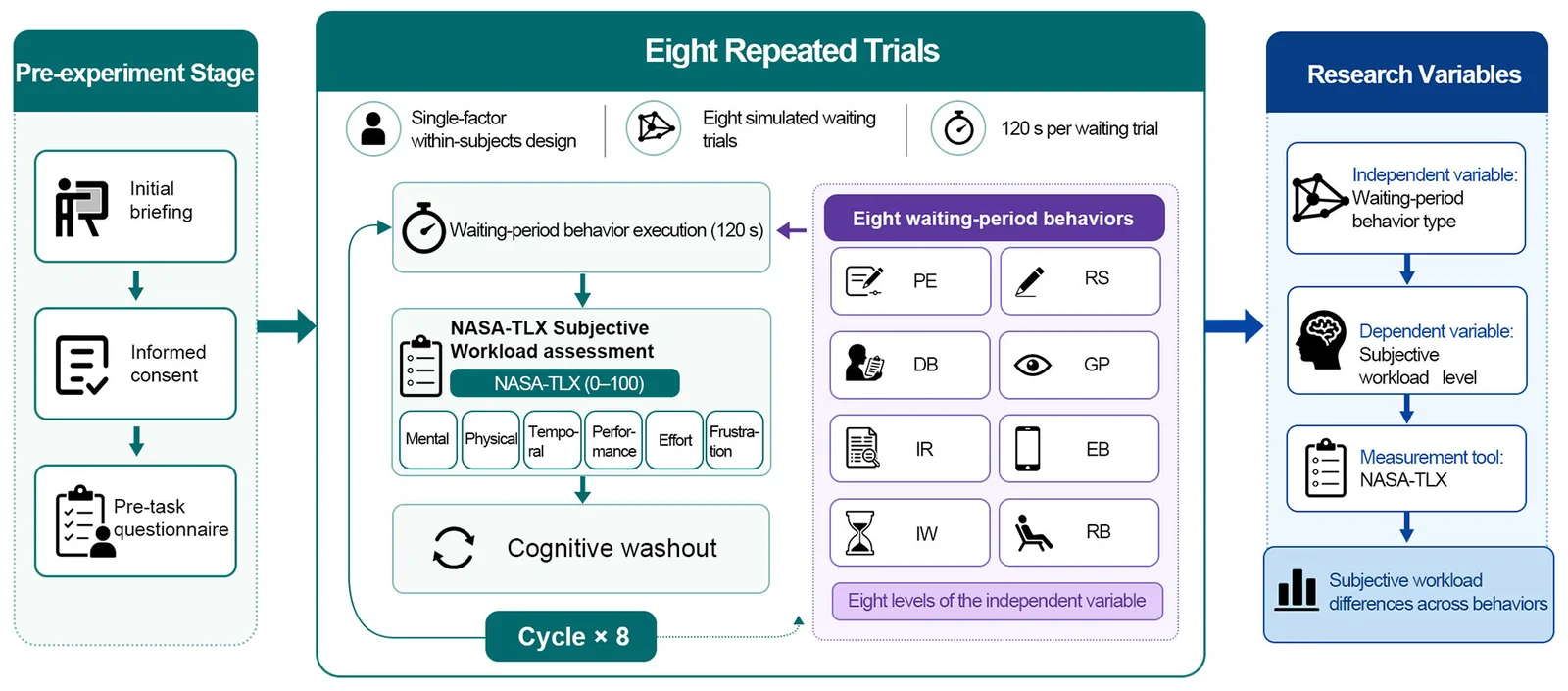
Procedure overview of Experiment 2.

#### 3.1.6 Data analysis

First, a one-way repeated-measures analysis of variance (ANOVA) was conducted to test for differences in subjective workload across the eight waiting-period behaviors, and the sphericity assumption was tested using Mauchly's test of sphericity. Given that NASA-TLX scores vary depending on task type and research context, their interpretation typically requires a comparative reference provided by similar tasks (Said et al., 2020; Hertzum, 2021). Therefore, this study did not use external absolute thresholds to classify workload levels. Instead, it used the eight behaviors examined in Experiment 2 as a common reference, ranked them based on their mean Raw TLX scores, and defined the top four and bottom four behaviors—based on the midpoint of the ranking—as those with relatively high and relatively low workload, respectively, within the current set of behaviors. This relative ranking was used to select representative behavioral conditions for the 2 × 2 design in Experiment 3; it does not imply that the eight behaviors naturally form two categories of workload with fixed thresholds.

In addition, based on the task-relevance classification results from Experiment 1, to establish four representative conditions—task relevance (high/low) × subjective workload (relatively high/low)—for subsequent analysis, this study further selected one behavior with a relatively high workload and one with a relatively low workload from each of the high- and low-task-relevance categories—namely, RS, DB, GP, and RB—and compared them using paired-sample *t*-tests, with the *p*-values from the four tests corrected using the Bonferroni method.

### 3.2 Results

#### 3.2.1 Subjective workload levels across different waiting-period behaviors

Mauchly's test of sphericity indicated that the assumption of sphericity was met, *W* = 0.231, *p* = 0.323. The one-way repeated-measures ANOVA showed a significant effect of waiting-period behavior on subjective workload scores (see Table 4), *F*_(7, 161)_ = 2.579, *p* = 0.015, and η*p*^2^ = 0.101. This indicates that there are significant differences in participants' subjective workload levels when performing different waiting-period behaviors. Based on the ranking of mean Raw TLX scores, PE, RS, IR, and GP occupied the top four positions and were classified as having relatively higher subjective workload within the current behavior set, whereas RB, IW, EB, and DB occupied the bottom four positions and were classified as having relatively lower subjective workload. This classification reflects the relative ordering of the eight behaviors within the context of the present study.

**Table 4:** One-way repeated-measures ANOVA results for NASA-TLX scores across eight waiting-period behaviors.

Behavior types	*M*	*SD*	*F*	*p*	*ηp* ^2^
PE	33.35	21.01	2.579	0.015^*^	0.101
RB	21.17	13.62			
RS	35.22	16.24			
IW	21.99	13.06			
IR	31.57	19.37			
EB	28.60	17.83			
DB	22.36	14.25			
GP	28.86	16.84			

^*^p < 0.05.

The paired comparisons involving the four representative behaviors selected for Experiment 3 showed that RS and GP both produced significantly higher subjective workload than DB and RB (see Table 5). All four comparisons—RS vs. DB, RS vs. RB, GP vs. DB, and GP vs. RB—were significant after Bonferroni correction, with adjusted *p*-values ranging from .018 to .045 and Cohen's *d*_*z*_ values ranging from 0.562 to 0.644.

**Table 5:** Paired-samples *t*-test results for subjective workload across the four representative waiting-period behaviors in Experiment 3.

Comparison	Mean difference	95% CI	*t*_(23)_	Bonferroni-adjusted *p*	Cohen's *d_*z*_*
RS–DB	12.87	[3.40, 22.34]	2.811	0.040^*^	0.574
RS–RB	14.05	[4.84, 23.26]	3.156	0.018^*^	0.644
GP–DB	6.51	[1.62, 11.39]	2.755	0.045^*^	0.562
GP–RB	7.69	[2.48, 12.90]	3.054	0.023^*^	0.623

^*^p < 0.05 after Bonferroni correction for the four comparisons.

Based on the task-relevance assessment from Experiment 1 and the subjective workload results from Experiment 2, RS, DB, GP, and RB were selected to represent four conditions: high task relevance × relatively higher workload, high task relevance × relatively lower workload, low task relevance × relatively higher workload, and low task relevance × relatively lower workload, respectively. The basis for their selection is presented in Table 6.

**Table 6:** Summary of the selection basis for the four representative waiting-period behaviors in Experiment 3.

Behavior	Task relevance score	Relevance classification	Raw TLX mean	Subjective workload classification	Representative condition
RS (Rapid sketching)	25.50	High	35.22	Higher	High task relevance × relatively higher workload
DB (Design brief review)	22.00	High	22.36	Lower	High task relevance × relatively lower workload
GP (Generation progress monitoring)	14.50	Low	28.86	Higher	Low task relevance × relatively higher workload
RB (Rest break)	8.00	Low	21.17	Lower	Low task relevance × relatively lower workload

### 3.3 Interim discussion

Different types of waiting-period behaviors exerted a significant influence on subjective workload. The results of Experiment 2 suggest that participants experienced different levels of subjective workload when performing different behaviors during the waiting period of AIGC-based image generation. These results suggest that waiting-period behaviors can be distinguished not only by task relevance but also by the subjective workload they elicit. According to Cognitive Load Theory, individuals have limited working memory resources available during task processing. Excessively high cognitive load may consume resources needed for subsequent information processing, whereas insufficient cognitive load may reduce task engagement and thereby impair problem-solving performance (Sweller, 1988).

## 4 Experiment 3: effects of task relevance and relative workload of AIGC waiting-period behaviors on creativity in conceptual design

Experiment 3 employed a behavior-based 2 × 2 conceptual framework. Based on the task-relevance classification from Experiment 1 and the subjective workload measurements and relative rankings from Experiment 2, four representative waiting-period behaviors—RS, DB, GP, and RB—were selected. These correspond to the four combinations of task relevance (high/low) × relative workload (higher/lower), and design creativity performance was compared across different conditions. Neither Experiment 3 nor its analysis plan was pre-registered; therefore, all inferential analyses in this experiment are reported as exploratory analyses.

### 4.1 Methods

#### 4.1.1 Participants

The study additionally recruited 60 graduate students majoring in design-related fields from Ningbo University in China as participants (age: *M* = 23.78, *SD* = 1.51), with a gender ratio of nearly 1:1. Prior to the experiment, the participants' design abilities were assessed and ranked based on the design works they submitted. The ranking was conducted by the same course instructor for all participants, who evaluated and ranked the design works submitted as course assignments. Subsequently, the 60 participants were ranked from highest to lowest in design ability, with every four participants forming a block, resulting in a total of 15 blocks. Within each block, the four participants were randomly assigned to one of the four behavioral conditions under this conceptual framework, with one participant assigned to each condition. Ultimately, each experimental condition included 15 participants, ensuring that the distribution of participants across the four conditions was as balanced as possible across different levels of design ability. This procedure constitutes a matched-block random assignment based on design ability ranking. The experiment was conducted at Ningbo University. All participants were required to sign informed consent forms before the commencement of the experiment and participated voluntarily, receiving a specified compensation upon the conclusion of the study. As no a priori power analysis was conducted, a sensitivity analysis was performed based on the achieved sample size. With α = 0.05, statistical power of 0.80, *N* = 60, four groups, and 1 degree of freedom for the effect test, the 2 × 2 between-subjects design had a minimum detectable effect size of Cohen's *f* = 0.368, indicating adequate sensitivity to effects in the moderate-to-large range or larger. According to APA ethical guidelines, the ethics committee of the institution where the first author is affiliated has approved this research.

#### 4.1.2 Creativity assessment

To minimize the potential influence of experimental condition information on scoring, this study invited five experts in the field of design to form a review panel. All valid concept sketches were anonymized and randomly sorted; the reviewers were unaware of the participants' experimental conditions (RS, DB, GP, or RB) when scoring. This study adopted a domain-specific creativity evaluation framework inspired by the relevant dimensions of the TTCT and incorporated design fixation to evaluate the creativity of conceptual designs across five dimensions: design fixation, fluency, flexibility, originality, and refinement (Jansson and Smith, 1991; Torrance, 1966). Specifically, design fixation, originality, and refinement were assessed using expert ratings on a 1–7 scale, whereas fluency and flexibility were derived from rule-based raw counts and linearly transformed to a 1–7 scale only for the calculation of the overall creativity score.

Design fixation was operationalized as the degree to which a proposal reused key features from the reference images. The five experts independently compared each valid design with both the initial example image and the same AI-generated image presented to all participants after the waiting period, rating each design on a structured 1–7 scale based on overall configuration, spatial relationships among components, manner of functional implementation, and distinctive morphological details. A score of 1 indicated no identifiable reuse of reference features, a score of four indicated partial reuse of key features while the overall design remained clearly distinct, and a score of seven indicated high similarity in multiple distinctive features as well as the overall configuration. For each proposal, the experts' mean rating was calculated separately for each of the two reference images, and the higher of the two mean ratings was taken as the proposal's fixation score. Subsequently, the average of all valid proposals submitted by each participant was calculated to obtain their raw design fixation score. A higher score indicates stronger design fixation.

The raw fluency score was the total number of valid design proposals submitted by participants within the specified time limit (Alabbasi et al., 2022). A proposal is counted as a separate entry only if it is recorded independently and expresses a complete, recognizable, and task-relevant solution concept. Separate entries can be identified by distinct sheets, numbering, or clear spatial separation. Scattered lines, content with unrecognizable design intent, or content unrelated to the task are not counted as valid proposals. Concepts that are similar but independently express complete solutions may be counted separately toward Fluency; different views, close-ups, textual descriptions, and adjustments to color, scale, lines, or local details within the same proposal were not counted repeatedly. Category membership was considered separately when calculating flexibility (Shah et al., 2003). The raw flexibility score was defined as the number of distinct idea categories represented across the valid design proposals, with each category counted only once regardless of how many proposals it contained. Dimension-level analyses of fluency and flexibility were conducted using their respective raw counts. Only when computing the overall creativity score were the raw counts for these two dimensions converted to a 1–7 scale using linear min–max normalization, with the full sample of 60 participants serving as the common reference set (Mazziotta and Pareto, 2022). The calculation formula was as follows:


$$\begin{array}{l} {\text{F}}_{\text{i}}^{*}\;=\;1\;+\;6\;\frac{{\text{F}}_{\text{i}}-{\text{F}}_{\min}}{{\text{F}}_{\max}-{\text{F}}_{\min}},\;{\text{L}}_{\text{i}}^{*}\;=\;1\;+\;6\;\frac{{\text{L}}_{\text{i}}-{\text{L}}_{\min}}{{\text{L}}_{\max}-{\text{L}}_{\min}} \end{array}$$

where *Fi* and *Li* denote participant *i*'s number of valid ideas and number of idea categories, respectively; *F*_min_, *F*_max_, *L*_min_, and *L*_max_ denote the minimum and maximum values observed across all 60 participants for the corresponding dimension; and Fi^*^ and Li^*^ are the transformed 1–7 scores.

Originality was used to assess the novelty and uniqueness of the design concepts, whereas refinement assessed the level of detail and completeness of their presentation. Both dimensions were rated on a structured 1–7 scale. For originality, a score of one indicated a conventional concept that was highly similar to common solutions; a score of four indicated the presence of some novel elements or combinations, although the overall concept remained broadly familiar; and a score of 7 indicated clear novelty and uniqueness, with the concept being clearly distinct from common solutions. For refinement, a score of one indicated a minimally developed proposal with limited information on key structures, functions, or design details; a score of four indicated that the main concept and functional features were represented with some supporting detail; and a score of seven indicated a highly detailed and comprehensively presented proposal in which the main structures, functions, and relevant design information were fully represented. For these expert-rated dimensions, scores of 2–3 and 5–6 represented transitional levels between adjacent anchor points.

Design fixation reflects the extent of reliance on existing reference features and the degree to which the design space is constrained. Higher fixation indicates greater dependence on reference features. Accordingly, its scoring direction is opposite to that of the other creativity dimensions (Jansson and Smith, 1991; Wadinambiarachchi et al., 2024). To ensure a consistent scoring direction across dimensions, the design-fixation score was reverse-scored according to the following formula when calculating the overall creativity score:


$$\begin{array}{l} D_{i}^{R}\;=\;8-D_{i} \end{array}$$

Here, *Di* denotes the raw design-fixation score, whereas *Di*^*R*^ denotes the reverse-scored design-fixation score. The five dimensions—reverse-scored design fixation, fluency, flexibility, originality, and refinement—were then combined using equal weights, with each dimension contributing 20% to the composite score. The overall creativity score for participant *i* was calculated as follows:


$$\begin{array}{l} C_{i}\;=\;\frac{D_{i}^{R}\;+\;F_{i}^{*}\;+\;L_{i}^{*}\;+\;O_{i}\;+\;E_{i}}{5} \end{array}$$

Here, Fi^*^ and Li^*^ denote the fluency and flexibility scores obtained through the linear min–max transformation described above, whereas *Oi* and *Ei* denote the mean expert ratings for originality and refinement, respectively. Apart from the 1–7 linear transformation applied to fluency and flexibility to place them on a common scale and the reverse scoring of design fixation, no further *z*-score standardization or other transformations were applied to the five dimensions before they were combined into the composite score. The overall creativity score therefore remained on a 1–7 scale, with higher scores indicating higher overall creative performance.

#### 4.1.3 Experimental design

Experiment 3 employed a behavior-based 2 × 2 conceptual framework comprising four between-subjects behavior conditions. Based on the task-relevance classification from Experiment 1 and the subjective workload results from Experiment 2, RS, DB, GP, and RB were selected to represent the four combinations of high task relevance × relatively higher workload, high task relevance × relatively lower workload, low task relevance × relatively higher workload, and low task relevance × relatively lower workload, respectively. Participants were randomly assigned to the four behavior conditions, with 15 participants in each condition. This framework was used to examine how differences among the four behavior conditions were patterned across the two theoretical dimensions. The dependent variable was design creativity.

#### 4.1.4 Materials

Experiment 3 used some of the materials from Experiment 2. To preserve the experiential features of the AIGC-assisted design process while ensuring that all participants were exposed to the same visual stimuli, a standardized AIGC interaction simulation interface was developed for this experiment. Through this interface, all participants experienced the same duration of image-generation waiting and viewed the same preset task-relevant AI-generated image after the waiting period.

#### 4.1.5 Procedure

The procedure for Experiment 3 was as follows (see Figure 6). Participants completed the experimental task using paper and pen within a simulated human–AI collaborative design process. In the main task, participants in all conditions received the same design brief. The conceptual design task was as follows: “Design a virtual avatar for a chatbot that communicates with users and conveys friendliness, intelligence, and interactivity.” This task was adapted from an experiment examining the effects of generative AI on design fixation and divergent thinking (Wadinambiarachchi et al., 2024). Before the ideation task, design fixation was induced by presenting participants with an example image. Participants then used the standardized AI-generation system developed specifically for this experiment to generate inspirational images. During the image-generation waiting period, they were instructed to perform the assigned waiting-period behavior for 2 min. After the waiting period ended, the system displayed the same preset AI-generated image related to the task theme to all participants.

**Figure 6:**
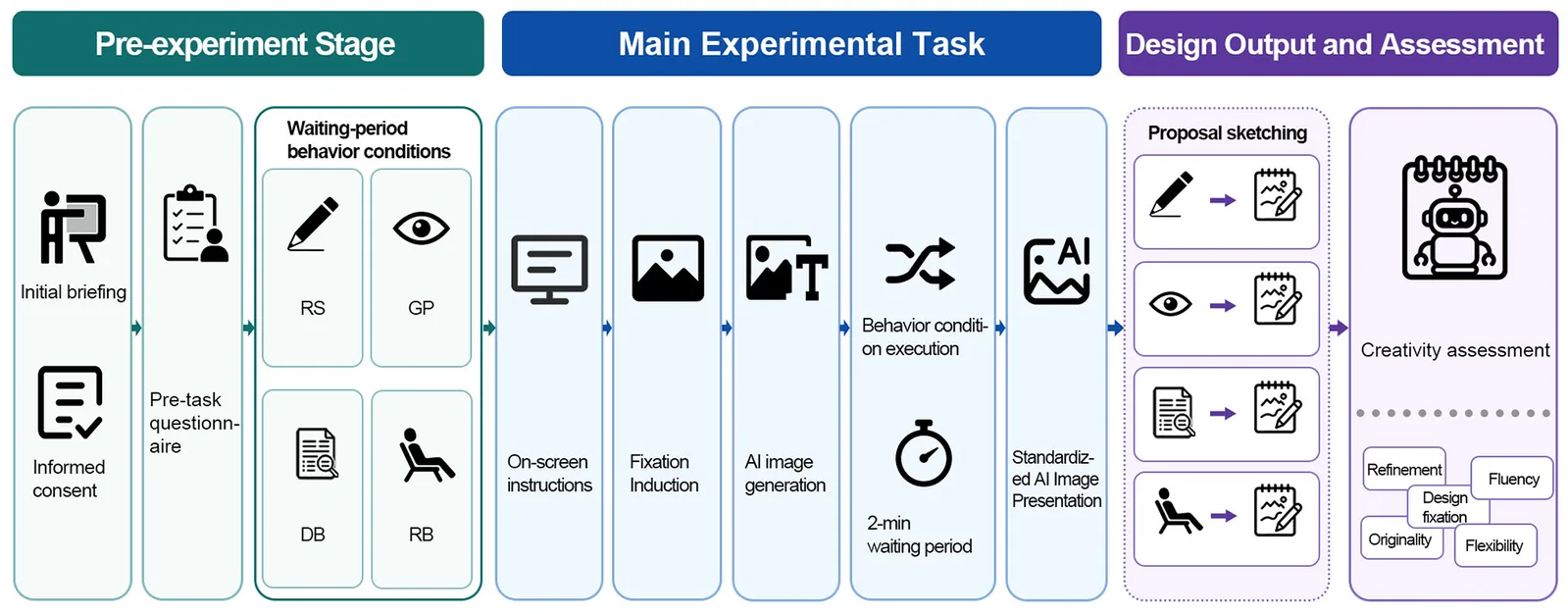
Procedure overview of Experiment 3.

Finally, participants had 20 mins to complete the design task. Previous studies have suggested that this duration is appropriate for maintaining focus while supporting both the quality and quantity of creative output (Vasconcelos and Crilly, 2016; Youmans, 2011). During this stage, participants were encouraged to present their design ideas through a combination of visual and textual elements.

### 4.2 Results

Because fluency and flexibility were derived from rule-based raw counts, inter-rater agreement was assessed only for the three expert-rated dimensions: design fixation, originality, and refinement (see Table 7). The overall creativity score was an equally weighted composite of these three expert-rated dimensions and the two rule-based count dimensions, rather than an independent overall judgment made by the experts; accordingly, no separate ICC was calculated for the composite creativity score. The ICC(A,1) values for design fixation, originality, and refinement were 0.594, 0.674, and 0.774, respectively, whereas the corresponding ICC(A,5) values were 0.880, 0.912, and 0.945. Because the final scores for these three dimensions were based on the mean ratings of the five experts, ICC(A,5) was taken as the primary reliability estimate. The results indicated a high level of agreement for the averaged expert ratings across all three dimensions.

**Table 7:** Intraclass correlation coefficient (ICC) results.

Rating dimension	ICC(A,1)	95% CI	ICC(A,5)	95% CI
Design fixation	0.594	[0.516, 0.671]	0.880	[0.842, 0.911]
Originality	0.674	[0.574, 0.767]	0.912	[0.871, 0.943]
Refinement	0.774	[0.695, 0.844]	0.945	[0.919, 0.964]

#### 4.2.1 Differences in design creativity on the task-relevance dimension based on generative-waiting-period behavioral conditions

Descriptive statistics show (see Table 8) that under high-task-relevance conditions, the overall creativity scores for the RS and DB conditions were *M* = 4.98, 95% CI [4.74, 5.23] and *M* = 5.07, 95% CI [4.66, 5.49], respectively; under low task-relevance conditions, the overall creativity scores for the GP and RB conditions were *M* = 3.77, 95% CI [3.33, 4.20] and *M* = 4.94, 95% CI [4.47, 5.42], respectively. Overall, design creativity scores under high task-relevance conditions exhibited a higher descriptive trend.

**Table 8:** Descriptive statistics of design creativity scores across the four experimental conditions.

Condition	*n*	*M*	*SD*	95% CI
RS (high relevance × relatively higher workload)	15	4.98	0.48	[4.74, 5.23]
DB (high relevance × relatively lower workload)	15	5.07	0.82	[4.66, 5.49]
GP (low relevance × relatively higher workload)	15	3.77	0.86	[3.33, 4.20]
RB (low relevance × relatively lower workload)	15	4.94	0.93	[4.47, 5.42]

Prior to conducting a two-way analysis of variance, the model's key statistical assumptions were tested. The Shapiro–Wilk test results indicated that the model residuals did not deviate significantly from a normal distribution, *W* = 0.980, *p* = 0.414. The Levene test for homogeneity of variances was also not significant, *F*_(3, 56)_ = 2.368, *p* = 0.080, suggesting that the assumption of homogeneity of variances was not violated. The two-way ANOVA results (see Table 9) showed that, under the current behavioral condition mapping, there was a significant difference in overall creativity scores between the high- and low-task-relevance conditions: *F*_(1, 56)_ = 10.864, *p* = 0.002, and η*p*^2^ = 0.162.

**Table 9:** Two-way ANOVA results for the effects of task relevance and subjective workload on design creativity scores.

Source	Sum of squares	df	Mean square	*F*	*p*	*ηp* ^2^
Task relevance	6.801	1	6.801	10.864	0.002^**^	0.162
Subjective workload	6.017	1	6.017	9.612	0.003^**^	0.146
Task relevance × subjective workload	4.461	1	4.461	7.126	0.010^**^	0.113

^**^p < 0.01. p values are rounded to three decimal places.

#### 4.2.2 Differences in design creativity on the subjective workload dimension based on generative-waiting-period behavioral conditions

Descriptive statistics also showed that design creativity scores were descriptively higher under the relatively lower workload conditions than under the relatively higher workload conditions. The two-way ANOVA further indicated that, under the current behavioral-condition mapping, overall creativity scores differed significantly between the relatively higher and relatively lower workload conditions, *F*_(1, 56)_ = 9.612, *p* = 0.003, and η*p*^2^ = 0.146.

#### 4.2.3 Interaction patterns between task-relevance and subjective workload

Further analysis revealed a significant interaction pattern between task relevance and subjective workload under the current behavioral-condition mapping, *F*_(1, 56)_ = 7.126, *p* = 0.010, and η*p*^2^ = 0.113. This indicates that the effect of subjective workload on design creativity is moderated by the task relevance of the behavioral task during the waiting period.

Simple-effects analyses showed that (see Table 10), under low task-relevance conditions, overall creativity scores were significantly higher in the relatively lower workload condition than in the relatively higher workload condition, mean difference = 1.179, 95% CI [0.60, 1.76], *t*_(56)_ = 4.080, Bonferroni-adjusted *p* = 0.0006, Cohen's *d* = 1.490. Under high task-relevance conditions, the relatively lower- and relatively higher workload conditions did not differ significantly, mean difference = 0.088, 95% CI [−0.49, 0.67], *t*_(56)_ = 0.305, Bonferroni-adjusted *p* = 1.000. On the other hand, under the relatively low workload condition, the difference between the low task-relevance group and the high task-relevance group was not significant: mean difference = −0.128, 95% CI [−0.71, 0.45], *t*_(56)_ = −0.443, and *p* = 1.000 after Bonferroni correction. However, under the relatively higher workload condition, the overall creativity score was significantly higher in the high-task-relevance group than in the low-task-relevance group; expressed as the low-minus-high task-relevance contrast, the mean difference was −1.219, 95% CI [−1.80, −0.64], *t*_(56)_ = −4.218, Bonferroni-adjusted *p* = 0.0004, and Cohen's *d* = −1.540.

**Table 10:** Simple-effects analysis of the interaction between task relevance and subjective workload.

Simple-effect comparison	Mean difference	95% CI	*t*_(56)_	Bonferroni-adjusted *p*	Cohen's *d*
Low task relevance: relatively lower –relatively higher workload	1.179	[0.60, 1.76]	4.080	0.0006^***^	1.490
High task relevance: relatively lower –relatively higher workload	0.088	[−0.49, 0.67]	0.305	1.000	0.111
Relatively lower workload: low –high task relevance	−0.128	[−0.71, 0.45]	−0.443	1.000	−0.162
Relatively higher workload: low –high task relevance	−1.219	[−1.80, −0.64]	−4.218	0.0004^***^	−1.540

^***^p < 0.001 after Bonferroni correction.

To directly compare the four specific waiting-period behavior conditions, RS, DB, GP, and RB were further treated as a four-level between-subjects factor, and a one-way ANOVA was conducted on overall creativity scores, followed by Tukey HSD *post-hoc* comparisons. The analysis revealed a significant difference in overall creativity across the four behavior conditions (see Table 11), *F*_(3, 56)_ = 9.201, *p* < 0.001, and η*p*^2^ = 0.330. Tukey HSD comparisons showed that the GP condition had significantly lower creativity scores than the RS, DB, and RB conditions (all *p* < 0.001), whereas no significant differences were observed among RS, DB, and RB (*ps* ≥ 0.971). At the level of specific behavior conditions, the primary between-condition pattern was therefore the lower overall creativity observed in GP relative to the other three conditions.

**Table 11:** Tukey HSD *post-hoc* pairwise comparisons of overall creativity scores across the four waiting-period behavior conditions.

Comparison	Mean difference (*I* – *J*)	95% CI	Tukey-adjusted *p*
RS–DB	−0.09	[−0.85, 0.68]	0.990
RS–GP	1.22	[0.45, 1.98]	< 0.001^***^
RS–RB	0.04	[−0.73, 0.81]	0.999
DB–GP	1.31	[0.54, 2.07]	< 0.001^***^
DB–RB	0.13	[−0.64, 0.89]	0.971
RB–GP	1.18	[0.41, 1.94]	< 0.001^***^

^***^p < 0.001 after Tukey HSD adjustment.

To examine whether differences in overall creativity are primarily driven by specific dimensions, this study further conducted a 2 × 2 two-way ANOVA, using fluency, flexibility, originality, refinement, and design fixation as dependent variables (see Supplementary Table S2). The results showed that task relevance was significant for fluency, originality, refinement, and design fixation; subjective workload was significant for flexibility, originality, refinement, and design fixation; and the interaction effect was significant for flexibility, originality, and refinement. Overall, differences in overall creativity involve multiple dimensions, but different dimensions exhibit distinct patterns of effects.

### 4.3 Interim discussion

According to the results of Experiment 3, creative performance across the four waiting-period behavior conditions showed a pattern of differences corresponding to task relevance and subjective workload. The 2 × 2 analysis revealed a significant interaction between task relevance and subjective workload under the current behavioral-condition mapping: under low task relevance, creativity scores were significantly lower in the GP condition than in the RB condition, whereas under high task relevance, no significant difference was observed between the RS and DB conditions. The four-level behavior analysis further showed that the primary between-group pattern in Experiment 3 was the lower creativity observed in GP relative to RS, DB, and RB. Overall, these findings indicate that the creativity differences observed across the current behavior conditions cannot be explained simply in terms of high vs. low subjective workload, but instead reflect the pattern associated with different combinations of task relevance and subjective workload.

## 5 General discussion

The waiting period before AI image generation is a critical stage that influences subsequent creative performance. Although this interval is relatively brief, it may elicit different behavioral choices, patterns of attention allocation, and levels of cognitive engagement, thereby influencing designers' cognitive state as they enter the subsequent creative stage.

The findings of this study suggest that behaviors during the AIGC generation waiting period can be understood from three interrelated functional perspectives. First, task continuity refers to the maintenance of current design goals and relevant cues during the waiting period, as reflected in the relatively high task relevance of both RS and DB. Second, cognitive expenditure concerns the use of limited cognitive resources by activities undertaken during the waiting period. Creative performance was lower in GP under the combination of low task relevance and relatively higher workload, whereas RS, despite also involving relatively higher workload, did not exhibit a comparable disadvantage. This contrast suggests that the cognitive consequences of the generation waiting period may depend not only on the magnitude of cognitive demand but also on whether the cognitive resources consumed remain directed toward the ongoing creative task. Third, temporary disengagement/incubation involves a temporary reduction in direct task processing. RB did not differ significantly from either RS or DB in creative performance, suggesting that moderate disengagement may also provide opportunities for attentional restoration or fixation release. The generation waiting period therefore cannot be reduced to a simple dichotomy between “continuous engagement” and “complete disengagement”; its potential value is better understood in terms of different configurations of task continuity, cognitive expenditure, and temporary disengagement/incubation.

From the perspective of AIGC-assisted design practice, the findings suggest that, within the current experimental context, maintaining task-related engagement during the waiting period while avoiding task-unrelated activities with high cognitive demand may represent a behavioral strategy worthy of attention. Examples include reviewing design goals, organizing preliminary ideas, and engaging in lightweight associative thinking about possible solutions. It should be noted, however, that the behaviors in Experiments 2 and 3 were induced through experimental instructions and performed for a fixed duration of 120 s. These practical implications therefore primarily reflect behavioral patterns observed under controlled experimental conditions and should not be directly equated with the effects of self-selected behaviors during real-world AIGC use.

## 6 Limitations and future research

Several limitations of the present study suggest directions for future research.

First, Experiment 3 has certain limitations in the operationalization of its conditions. The four combinations of the 2 × 2 structure were each represented by a single specific behavior—RS, DB, GP, and RB—so the current results cannot yet fully distinguish the respective contributions of task relevance, subjective workload, and behavior-specific characteristics. Future research could include multiple representative behaviors within each combination or manipulate the two theoretical dimensions more independently.

Second, this study also has certain limitations regarding manipulation checks and the measurement of psychological responses. After Experiment 3, participants' immediate evaluations of the task relevance and subjective workload of the four assigned behaviors were not collected again. Whether participants performed the assigned behaviors was mainly confirmed through on-site observation, but no video or interaction records were retained for subsequent verification. Future experiments could include prespecified post-experimental manipulation checks and establish explicit criteria for behavioral compliance. In addition, the NASA-TLX results obtained in this study reflect participants' self-reported subjective workload and were not complemented by physiological measures. Future research could combine subjective reports with secondary-task performance, eye tracking, pupillary changes, or other process measures to directly examine these psychological mechanisms and their roles in the effects of waiting-period behaviors on design creativity.

Third, the ecological applicability of the findings is also constrained by differences between the observational and experimental contexts. Experiment 1 recorded behaviors that participants autonomously selected during naturally occurring AIGC waiting periods, whereas Experiments 2 and 3 required participants to perform assigned behaviors continuously for 120 s according to experimental instructions. Actively choosing a behavior during a natural waiting period and performing the same behavior under instruction may involve different behavioral motivations, attentional states, and levels of engagement. The fixed 120-s waiting duration also cannot capture shorter or longer generation delays encountered during actual AIGC use. Experiment 3 examined only the immediate creativity outcomes following a single waiting-period behavior and did not address behavioral adaptation, strategy adjustment, or the persistence of effects across repeated rounds of human–AI collaboration. Future research could compare self-selected and experimentally assigned waiting-period behaviors, systematically manipulate waiting duration, and adopt naturalistic, multi-round interaction, or longitudinal designs to examine the dynamic changes in waiting-period strategies and their effects during human–AI collaborative design.

Finally, the sample composition and task settings limit the applicability of the findings. All three experiments recruited students from design-related programs at Ningbo University, China. Experiment 1 included both undergraduate and graduate students, whereas Experiments 2 and 3 included graduate students only, and each experiment employed only one design task. Given that creativity is domain-specific and that cultural background may influence creative output and its evaluation (Baer, 2012; Niu and Sternberg, 2001), the current findings are primarily applicable to the educational, cultural, and task contexts examined in this study. Future research could include professional designers and participants from a wider range of cultural backgrounds and replicate the findings across multiple design tasks.

## 7 Conclusion

Through three experiments, this study examined how behavioral differentiation during the AIGC generation waiting period relates to creativity in conceptual design. The results indicate that the generation waiting period is not merely a form of technical latency, but a processual interval involving multiple identifiable behavioral patterns. These behaviors differ in task relevance and subjective workload and correspond to differences in subsequent creative performance in conceptual design.

Based on these findings, this study further suggests that research on AIGC-assisted conceptual design should not focus solely on prompt quality, the visual quality of generated images, or the evaluation of generated outputs. It should also attend to how designers organize their behaviors during the generation waiting period. For design practice, a more appropriate waiting-period strategy is neither complete disengagement from the task nor engagement in high-load task-irrelevant activities. Rather, designers should moderately regulate their cognitive engagement while maintaining task relevance, so as to preserve the continuity of design thinking and reduce task-irrelevant cognitive expenditure. Theoretically, this study distinguishes three interrelated functional aspects of the AIGC generation waiting period: task continuity, cognitive expenditure, and temporary disengagement/incubation. On this basis, it proposes that the cognitive consequences of the waiting period depend not only on the magnitude of cognitive demand but also on whether the cognitive resources consumed remain directed toward the ongoing creative task. This provides a more specific explanatory framework for understanding why different waiting-period behaviors correspond to different levels of creative performance.

## Data Availability

The raw data supporting the conclusions of this article will be made available by the authors, without undue reservation .
