# Supplementary material for: Behavioral differentiation during the AIGC image generation waiting period: effects on creativity in conceptual design
Source: Front Psychol. 2026 Oct 12;17:1924155. doi: 10.3389/fpsyg.2026.1924155 (PMC13696403; doi:10.3389/fpsyg.2026.1924155)
Supplement: Supplementary file 1 [file Supplementary_File_1.docx]

Supplementary Material

# 1 Supplementary Figures and Tables

## 1.1 Supplementary Tables

Supplementary Table 1 presents the 17 candidate waiting-period behavior categories developed in Experiment 1, together with their operational definitions and additional coding instructions. The final column indicates whether each category was retained in the 11-behavior core set after the two-indicator screening based on total occurrence frequency and cumulative duration. The additional coding instructions specify key boundary cases used to distinguish conceptually similar behaviors.

**Supplementary Table 1.** Candidate AIGC waiting-period behaviors: operational definitions, coding criteria, and core-behavior retention

| **Candidate behavior** | **Code** | **Operational definition** | **Additional coding instructions** | **Core behavior retained** |
| --- | --- | --- | --- | --- |
| Reference information retrieval | IR | The participant actively searches for and browses external information related to the current design task, including the design object, competing products, structures, use scenarios, or design styles. A segment is coded here when a clear task-related search target is observable and the participant proceeds to open, inspect, or trace specific reference content. | Task-related viewing confined to the search-results list is coded as task-related shallow search; browsing without a clear task goal is coded as unstructured web browsing and switching. | Yes |
| Prompt editing | PE | The participant independently composes or substantively modifies the prompt currently used for image generation, including adding, deleting, replacing, restructuring, or refining keywords, phrases, or constraints. | Copying or pasting an LLM-generated prompt unchanged is not coded as PE; coding begins at the first substantive independent modification. | Yes |
| Generation progress monitoring | GP | While the image is still being generated, the participant checks the generation process or status update by continuously watching the generation area, intermittently checking status information, or actively refreshing/reloading the page. | Applies only before generation is complete; post-generation viewing is coded as generated-result browsing. | Yes |
| Social messaging interaction | SM | The participant checks, types, sends, or exchanges direct chat messages unrelated to the current design task in an instant-messaging application. | Browsing social feeds such as WeChat Moments is coded as entertainment content browsing. | Yes |
| Design brief review | DB | The participant reopens, rereads, or reviews the current design-task instructions, including the design topic, task goals, and specified constraints such as dimensions, users, scenarios, or functional requirements. |  | Yes |
| Rest break | RB | The participant stops the current task activity and displays observable restorative or relaxing actions or postures, such as closing the eyes, stretching, or clearly leaning back. | Eyes-open waiting in a normal seated posture without clear restorative behavior is coded as idle waiting. | Yes |
| Rapid sketching | RS | The participant rapidly uses pen and paper or another drawing tool to depict the overall product outline, local structure, component relationships, or alternative forms related to the current design task in order to externalize a design idea. |  | Yes |
| Idle waiting | IW | During the generation wait, the participant stops explicit task operations, remains eyes open in a normal waiting posture, or lets the gaze drift without a fixed task target, while no other identifiable activity is observed. | Eye closure, stretching, or marked leaning back is coded as a rest break. | Yes |
| Unstructured web browsing and switching | UW | Without a clear task-related search goal, the participant casually switches among browser tabs, webpages, search results, or design websites, scrolls, or briefly inspects content. | Behavior with a clear task-related search goal is not included; shallow task-related viewing of results pages is coded as task-related shallow search. | Yes |
| Entertainment content browsing | EB | The participant browses, scrolls through, or watches entertainment, lifestyle, leisure, social-feed, or short-video content unrelated to the current design task. | Direct-chat message checking or replying is coded as social messaging interaction. | Yes |
| AI-assisted prompt generation | AP | In an external large-language-model interface, the participant asks the model to generate or revise a prompt for subsequent image generation, reads the model output, and may copy and transfer the generated prompt unchanged to the image-generation interface. | Unchanged copying/pasting remains in this category; coding switches to PE from the first substantive independent modification. | Yes |
| Generated-result browsing | GRB | After image generation is complete, the participant views one or more generated outputs by enlarging, scrolling through, continuously inspecting, or comparing them. | Applies only after generation is complete; status checking during generation is coded as generation progress monitoring. | No |
| AIGC platform function and parameter exploration | APE | The participant actively opens, inspects, or tries functions, menus, buttons, parameter settings, or parameter combinations on an AIGC image-generation platform in order to understand available operations or parameter effects. |  | No |
| Task-related silent pause | TSP | The participant stops overt keyboard, mouse, or pen-and-paper operations and remains briefly silent, with the gaze sustained on the current sketch, design brief, or nearby work area. | A static posture alone is insufficient; retrospective information is required to confirm task relevance. | No |
| Non-task oral conversation | NTC | During the generation wait, the participant engages in identifiable oral conversation with the experimenter or another person that is unrelated to the current design task or experimental operations. |  | No |
| Self-grooming and posture adjustment | SPA | The participant's primary observable activity consists of self-grooming or posture-adjustment actions such as touching the face, rubbing the eyes, adjusting clothing or hair, or changing the sitting posture. |  | No |
| Task-related shallow search | TSS | The participant uses search terms directly related to the current design task and views the search-results list, but remains at the results-page level without opening specific results, reading them in depth, or tracing further information. | Opening and inspecting specific reference content is coded as reference information retrieval; browsing without a clear task goal is coded as unstructured web browsing and switching. | No |

**Note.** Yes = retained in the final core-behavior set; No = not retained. Boundary cases were coded according to the observable action, object of interaction, and activity content. retrospective information was used when the behavioral direction could not be determined from the recordings alone.

Supplementary Table S2 reports separate 2 × 2 ANOVAs for the five creativity dimensions in Experiment 3, examining task relevance, subjective workload, and their interaction for fluency, flexibility, originality, refinement, and design fixation.

**Supplementary Table S2.** Two-way ANOVA results for the five creativity dimensions in Experiment 3

| **Creativity dimension** | **Task relevance** | **Subjective workload** | **Interaction** |
| --- | --- | --- | --- |
| Fluency | *F* = 9.113, *p* = 0.004, ηp² = 0.140 | *F* = 3.492, *p* = 0.067, ηp² = 0.059 | *F* = 2.500, *p* = 0.119, ηp² = 0.043 |
| Flexibility | *F* = 2.314, *p* = 0.134, ηp² = 0.040 | *F* = 8.434, *p* = 0.005, ηp² = 0.131 | *F* = 5.527, *p* = 0.022, ηp² = 0.090 |
| Originality | *F* = 10.848, *p* = 0.002, ηp² = 0.162 | *F* = 6.456, *p* = 0.014, ηp² = 0.103 | *F* = 6.456, *p* = 0.014, ηp² = 0.103 |
| Refinement | *F* = 16.358, *p* < 0.001, ηp² = 0.226 | *F* = 11.204, *p* = 0.001, ηp² = 0.167 | *F* = 9.847, *p* = 0.003, ηp² = 0.150 |
| Design fixation | *F* = 6.325, *p* = 0.015, ηp² = 0.101 | *F* = 6.603, *p* = 0.013, ηp² = 0.105 | *F* = 3.662, *p* = 0.061, ηp² = 0.061 |

**Note.** All *F* tests had df = (1, 56). Fluency and flexibility were analyzed using raw counts; originality and refinement used the mean ratings of the five experts; design fixation used the original fixation score (higher scores indicate stronger fixation). ηp² = partial eta squared.
